# Effectiveness of transcutaneous electrical nerve stimulation in management of neuropathic pain in patients with post traumatic incomplete spinal cord injuries

**DOI:** 10.12669/pjms.345.15659

**Published:** 2018

**Authors:** Amir Zeb, Aatik Arsh, Sher Bahadur, Syed Muhammad Ilyas

**Affiliations:** 1 Amir Zeb, PPDPT, MSPT. Senior Physical Therapist, Paraplegic Center, Peshawar, Pakistan; 2 Aatik Arsh, DPT, MSPT. Physical Therapist, Paraplegic Center, Peshawar, Pakistan; 3 Sher Bahadur, MPH. Senior Research Officer Rehman Medical College, Paraplegic Center, Peshawar, Pakistan; 4 Syed Muhammad Ilyas, MSPT. Chief Executive Officer, Paraplegic Center, Peshawar, Pakistan

**Keywords:** Neuropathic pain, Pain management, Physiotherapy, Spinal cord injury, TENS

## Abstract

**Objective:**

To determine the effectiveness of Transcutaneous Electrical Nerve Stimulation (TENS) in management of neuropathic pain in post-traumatic incomplete spinal cord injury patients.

**Methods:**

A quasi-experimental study was conducted at from January 2017 to June 2017 at Paraplegic Center Hayatabad, Peshawar. Total 60 incomplete spinal cord injured patients with diagnosis of neuropathic pain were subjected to high frequency TENS of 80 HZ. One session was of 45 minutes while there were two sessions per day. TENS was applied for four days in a week and all patients were followed for eight week duration. Pain intensity was measured by using VAS (Visual analogue scale).

**Results:**

Mean pain intensity on VAS at baseline was 6.45 which was decreased to 4.77 post intervention at day-1 while it was decreased to 3.48 at day-4 of week one. After application of TENS for 8 weeks, mean pain intensity was decreased to 2.80 ± 1.74. During the consecutive sessions of the TENS application, the pain intensity decreases in a linear fashion and there were significant difference (p<0.05) between pre and post treatment sessions.

**Conclusion:**

TENS is useful and safe adjuvant in spinal cord injury patients for the management of neuropathic pain.

## INTRODUCTION

Spinal Cord Injury (SCI) is a devastating disability known to human kind.^1^ Despite the fact that SCI is associated with incredible costs and human sufferings, yet exact statistics of SCI are not available in majority of developing countries including Pakistan.^2^ The only available literature related to SCI in Pakistan is in the form of small single center based retrospective surveys.^3^-^8^

Pain is a common complication after SCI with prevalence of 18 to 96%, and almost 30% of these pain are diagnosed as neuropathic pain.^9^ The intensity of the pain varies amongst these patients and it has been reported that 77.7% of patients with spinal cord injuries have moderate to severe pain.^10^ Neuropathic pain mostly starts soon after injury and can continue for the rest of the patient’s life. The consequences of long term pain have often been associated negatively with the outcomes of rehabilitation.^11^ Neuropathic pain is usually managed with a variety of pharmacological and non-pharmacological therapies^12^ Among non-pharmacological treatments Transcranial Electrical Stimulation (TES), acupuncture, massage therapy and Transcutaneous Electrical Nerve Stimulation (TENS) are some of the frequently used modalities/techniques for relieving neuropathic pain in SCI patients. TENS is one of the commonly used physical therapy modality for the management of pain in Pakistan.^13^-^15^ In some clinical trial it was found that TENS had positive effects on management of pain in patients with SCI.^10^ However, other studies on the efficacy of TENS in relieving neuropathic pain in SCI patients have not shown any effects and/or shows contradictory outcomes.^10^,^16^,^17^ Due to controversy in literature, there was a dire need to conduct this study in order to determine the effectiveness of TENS in management of neuropathic pain in patients with post traumatic incomplete spinal cord injuries.

## METHODS

This quasi experimental study was conducted from January 2017 to June 2017 at Paraplegic Center Hayatabad, Peshawar. Paraplegic center Peshawar is second to none in the country providing comprehensive rehabilitative services to patients with spinal cord injuries.

A total of 60 patients with incomplete spinal cord injuries with diagnosis of neuropathic pain and age 20-60 years were recruited in the study using consecutive sampling technique. Complete spinal cord injury patients and/or those with complications e.g. pressure sores, fractures etc. were excluded.

TENS with high frequency of 80 HZ was applied to patients of SCI. Time for one session was 45 mints, while there were 2 sessions per day i.e. morning and evening session. TENS was applied for four consecutive days i.e. Monday, Tuesday, Wednesday, and Thursday. Each and every patient who entered into the study was followed for 8 weeks. Pain intensity was measured by using VAS before and after the application of TENS. Comparison between pre and post interventional scores was completed to see whether improvement occurred or not. Ethical approval was obtained from institutional ethical review committee of Paraplegic center, Peshawar.

## RESULTS

A total of 60 subjects with mean age 52.64±0.48 (ranged from 20-60 years) participated in the study, out of whom 75% (n=45) were male and 25% (n=15%) were female. Majority of the participants (71.7%, n=43) were married while the rest of participants (28.3%, n=17) were single. All the patients were from Khyber Pakhtunkhwa and were Pashto speaking. Most of the participants were laborer (55.0%, n=33), 13 (21.7%) participants were House wives while rest of the participants (23.3%, n=14) were having other professions. Forty six (76.7%) participants had thoracic paraplegia, 11 (18.3%) patients had lumbar paraplegia while 3 (5.0%) patients had cervical tetraplegia.

Mean pain intensity on VAS at baseline was 6.45±1.09 which were decreased to 4.77±1.52 post intervention at day-1 of week 1. The mean decreased in the pain intensity was seen through the array of intervention. The mean pain intensity was decreased to 3.48±1.91 at day-4 of week-1. During the consecutive sessions of TENS application, the pain intensity decreases in a linear fashion and there were significant difference (p<0.05) between pre and post treatment sessions. (Table-I)

**Table-I:** Comparison of Pain at pre and post treatment at different days of Week 1 and Week 8.

Pain at pre and post treatment		Mean ± SD	P-Value
Week 1, Day-1,	D1Pre	6.45 ± 1.09	<0.001
	D1Post	4.77±1.52	
Week 1, Day-2	D2Pre	5.96±1.20	< 0.001
	D2Post	4.06±1.57	
Week 1, Day-3	D3Pre	5.63±1.39	< 0.001
	D3post	3.88±1.99	
Week 1, Day-4	D4pre	5.37±2.00	< 0.001
	D4post	3.48±1.91	
Week 8, Day-1,	D1Pre	4.21± 1.78	<0.001
	D1Post	2.99 ± 1.33	
Week 8, Day-2	D2Pre	3.92±1.69	< 0.001
	D2Post	2.92 ±1.27	
Week 8, Day-3	D3Pre	3.41±1.52	< 0.001
	D3post	2.91 ± 1.52	
Week 8, Day-4	D4pre	3.10±1.42	< 0.001
	D4post	2.80 ± 1.74	

After application of TENS for 8 weeks, mean pain intensity at day 1 of week 8 was 2.99 ± 1.33 which was decreased to 2.92±1.2 on day-two and 2.91±1.5 on day three while 2.80±1.7 on day-4. There was significance difference (p<0.05) in pain intensity at pre and post intervention of each session. (Table-I)

## DISCUSSION

SCI is associated with various degree of sensory and motor dysfunctions.^18^ Neuropathic pain is common among spinal cord injury patients which can be managed both through pharmacological as well as Non-Pharmacological interventions.

Using the Quasi experimental design this study was aimed to determine the effectiveness of TENS in management of neuropathic pain in patients with post traumatic incomplete SCI patients. Result indicated that mean decreased in the pain intensity was seen through the array of intervention. During the consecutive sessions of the physical therapy the pain intensity decreased in a linear fashion and there are significant difference (p<0.05) between pre and post treatment session. Following TENS in patients with post Traumatic incomplete spinal cord injuries, mean pain score at week week-8 and day-4 was decreased to 2.80 which reveals a marked decreased in pain intensity. These results are consistent with a randomized control study which reveals that there was a marked decreased in the pain intensity after each consecutive intervention (application of TENS).^17^ Results indicates that after each session the pain decreases to a significant level (p<0.05). The results of the present study are in consistence with studies conducted by Norrbrink C et al. and Celik EC et al. who found that TENS complement pharmacological treatment in patients with SCI and neuropathic pain.^9^,^17^ From present study it was meticulous to see the low pain score (3.78) at base line of week-8, as it was 6.45 at week-1 and day-1. Similar finding was also reported by Kilinc M et al. who compared pre- and post TENS treatment for patients with peripheral (PNP) or central neuropathic pain (CNP). At the beginning of the trial, the minimal, maximal, mean, and current pain intensities were similar between the CNP and PNP groups. Post-treatment pain intensity values were significantly lower than pre-treatment values in both groups (p < 0.05).^19^-^20^

Certain adverse affects of TENS has been reported in literature.^17^ For example, high intensity TENS can cause rashes and local tingling sensations. 20 However these adverse reactions are minimal as compared to its beneficial effects. Though in current study patients were followed for 8 weeks, however there some studies reported relapse of neuropathic pain in spinal cord injury patients.^21^-^23^ There is possibility of relapse of neuropathic pain in participants of current study, therefore proper long term follow up is advised to all participants. TENS is primarily aimed to provide a degree of symptomatic pain relief by exciting sensory nerves.^21^-^22^ The effectiveness of TENS varies with the clinical pain being treated, but research suggest that when used ‘well’ it provides significantly greater pain relief than a placebo intervention.^23^

Despite the fact that current study is first of its kind conducted in Pakistan which reported effectiveness of TENS in managing neuropathic pain in spinal cord injury patients, however it has some limitations. First of all, its design was quasi-experimental so the results of current study can be affected by confounding variable. Secondly sample size of current study was small due to which generalizability of the results of current study is questionable.

## CONCLUSION

TENS is useful and safe adjuvant in spinal cord injury patients for the management of neuropathic pain. A consistence and long term rehabilitation with TENS in patients with post Traumatic incomplete SCI is useful.

### Authors’ Contribution

**AZ**: Concept and study design, literature search and literature review, Acquisition of data.

**AA:** Acquisition of data, drafting the manuscript.

**SB:** Analysis & interpretation of data.

**SM:** Critical revision, final approval of the version to be published.
